# Developing a dissemination and implementation research agenda for aging and public health: The what, when, how, and why?

**DOI:** 10.3389/fpubh.2023.1123349

**Published:** 2023-02-06

**Authors:** Paul A. Estabrooks, Russell E. Glasgow

**Affiliations:** ^1^Department of Health and Kinesiology, College of Health, University of Utah, Salt Lake City, UT, United States; ^2^ACCORDS Dissemination & Implementation Science Program and Department of Family Medicine, University of Colorado Anschutz Medical Campus, Denver, CO, United States

**Keywords:** translational sciences, knowledge translation (KT), dissemination & implementation research, reach, effectiveness, adoption, implementation and maintenance (RE-AIM)

## Introduction

To improve the uptake of evidence-based interventions in the fields of aging and public health there have been calls to apply the methods, models, and measures of dissemination and implementation science (DIS) (1, 2). DIS may be defined as the scientific study of the strategies and mechanisms by which research evidence is adopted, applied, and sustained in community or clinical settings to improve outcomes for a specified population (3, 4). Early work in DIS focused on expanding the reporting of outcomes. This translated into including, but moving beyond efficacy or effectiveness, when testing interventions to improve health outcomes and balancing internal and external validity in the development and testing of new interventions (5). In one early framework, these expanded outcomes were initially summarized by our research team using reach, effectiveness, adoption, implementation, maintenance (RE-AIM) dimensions (5, 6). Within RE-AIM, multi-leveled dissemination outcomes were operationalized at the level of the population intended to benefit (i.e., reach) as well as the staff, settings, and systems (i.e., adoption) intended to deliver an intervention (7). At each level of dissemination, researchers were encouraged to address representativeness, engage the populations and systems that could most benefit, and advance health equity (8, 9). Similarly, implementation outcomes were operationalized within RE-AIM at the staff, setting, and system levels to include the degree to which an intervention was delivered as intended (i.e., implementation), the costs associated with implementation, adaptations made, and the potential for sustainability (i.e., organizational level maintenance).

In addition to expanding outcomes, understanding context is a key aspect of DIS (10, 11). Contextual factors related to DIS outcomes provide constructs that can act as moderators, mediators, or mechanisms of success (4, 12). Indeed, the field has seen a proliferation of theories, models, and frameworks to provide systematic approaches to understand the relationships between contextual factors and outcomes (13–15). For example, the Practical, Robust, Implementation, and Sustainability Model (PRISM) provides constructs multi-level constructs of potential beneficiaries (e.g., economic status; compatibility of intervention with lifestyle) and potential implementers (e.g., expertise; complexity of intervention implementation); implementation and sustainability infrastructure (e.g., structured communication channels); and external environmental factors (e.g., community resources to support or inhibit dissemination and/or implementation) (16, 17). Each of these contextual constructs, when tied to a specific RE-AIM outcome, can be used to map strategies to improve outcomes that can be tailored to address contextual moderators or designed to leverage contextual mediators or mechanisms that lead to success (16, 18).

Over the past 2–3 years there have been several articles articulating how DIS can be applied to aging and health issues (1, 9, 19, 20). Of particular relevance is a paper by Carpenter et al. (19) that discusses how addressing DIS outcomes, moderators, mediators, and mechanisms using the Standards for Reporting Implementation Studies (StaRI) can be applied to advance DIS and aging research. The STaRI guidelines summarize key DIS issues under the various sections of a manuscript for reporting on DIS studies. For example, in the introduction, identification of the DIS theory or framework used is recommended while in the methods section clear operational definitions of the implementation context, outcomes, and economic evaluation are encouraged. The results and discussions sections are recommended to include information on fidelity to protocol, intervention adaptions, and generalizability to other typical clinical or community settings.

## Developing a DIS research agenda for aging and public health

Several researchers have developed guidance and recommendations about advancing DIS. To develop successful projects and outcomes, Kilbourne et al. (21) recommended the use of a conceptual model, collaborative methods (e.g., development of a shared agenda, implementation strategies, adaptation recommendations with key system partners), and focusing on building system capacity and a business case for sustained implementation. Other recommendations for advancing DIS include using mixed methods to capture important contextual and systems factors that may not be quantifiable (22, 23) and pragmatic approaches to maintain a focus on generalizability and usability of implementation strategies and outcomes relevant to typical clinical and community settings (24). In addition, to these recommendations, we propose the following areas for DIS in aging and public health.

### Focus on the how, what, when and why of dissemination and implementation

Early DIS often focused only on documenting the achievement of implementation (e.g., RE-AIM) outcomes. *Active for Life*, a multi-site project promoting physical activity in older adults is a good example. The primary focus was on determining if evidence-based physical activity programs could be delivered in typical community settings and demonstrate effectiveness (25, 26). Process evaluation also demonstrated that, possibly due to the collaborative nature of the multi-site trial, there was high implementation fidelity across communities and that communities adapted the interventions to improve fit with delivery settings (27). Studies like *Active for Life* were critical in addressing external validity and effectiveness, and set the stage for current DIS in aging to focus on understanding not only if dissemination and implementation outcomes can be achieved, but also *on understanding how those outcomes can be achieved by monitoring what strategy was used, and when, in the implementation process as well as analyzing why the outcome occurred by examining prespecified mechanism(s) or mediator(s)*.

**Figure 1:**
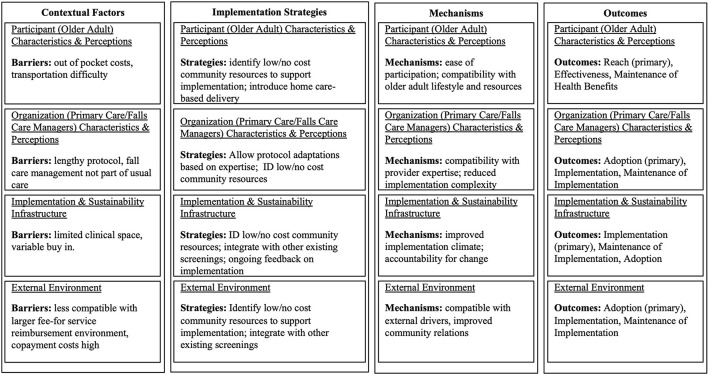
An example application of dissemination and implementation science in aging research: STRIDE, a national study to prevent fall related injuries.

Recently, Implementation Research Logic Models have been introduced as an example of how to better support DIS researchers and clinical or community partners to conceptualize and test the how, what, and why of dissemination and implementation outcomes (28). This approach encourages the use of theory to characterize contextual factors that can be used to (a) determine barriers and facilitators related to achieving DIS outcomes, (b) develop context-specific implementation strategies, and (c) identify potential mechanisms and mediators of change that (d) explain if and how changes in DIS outcomes occur as a result of an implementation strategy. We developed Figure 1 as a simplified example of how aging researchers could apply DIS using information from an excellent article on the Strategies to Reduce Injuries and Develop Confidence in Elders (STRIDE) pragmatic trial of an intervention for Falls Care Managers to reduce fall-related injuries in older adults (29).

To orient readers to the Figure each row reflects a path model that begins with barriers, strategies to overcome barriers, mechanisms, and outcomes relative to theory-based contextual factors—in this case using PRISM. The STRIDE investigative team reported on qualitative data they used to identify contextual factors that could inhibit intervention implementation and on strategies used across sites to address those factors. For ease of presentation, we only focused on barriers in the figure and linked barriers to reported strategies and then identified potential mechanisms based on PRISM contextual factors. Finally, the Figure identifies the primary DIS outcome that is most likely to change in response to the implementation strategy through the proposed PRISM mechanisms. Of note, implementation research logic models are prone to reductionism and our example uses a reductionist approach for simplicity. However, we also demonstrate that the barrier-strategy-mechanism-outcome link can be very complex with a single strategy, in part, addressing several implementation barriers (e.g., identify low/no cost community resources to support implementation) or conversely several DIS strategies may be needed to address a single barrier. Further, strategies often do not work through a single mechanism and a single mechanism is typically not responsible for a single DIS outcome. As such, we recommend the use of practical tools, such as logic models, to map out proposed relationships, develop hypotheses, and guide trials, but also to avoid oversimplification of the context-strategy-mechanism-outcome relationships.

### Acknowledge and address context and adaptations as dynamic factors

Related to the recommendation to avoid oversimplification and reductionism, relevant and active areas of DIS for aging and public health researchers include addressing multi-level contextual factors and adaptation. While public health has historically focused on multiple socio-ecological levels and multiple determinants of health (30), DIS has focused more specifically on key components of context (e.g., implementation infrastructure related to available pragmatic implementation feedback loops) and how the evidence-based programs align with key aspects of context (31). One of the central tenets of DIS is that context is not static, but changes over time, sometimes very rapidly as was seen during the initial (and ongoing) COVID-19 pandemic (11). Understanding, tracking and adapting to contextual changes undergirds DIS and illustrates how it is different than other types of health outcomes research (8, 11, 32).

Adaptations and the balance between evidence-based program implementation fidelity and context specific changes (e.g., tailoring) is critical for implementation success and sustainability (33, 34). DIS posits that fidelity should be to core functions or principles rather to a rigid protocol, and that adaptions to clinical context and conditions may also be necessary (35, 36). One DIS approach that is broadly applicable and often more intuitive than other approaches for community and clinical partners is that of *form* and *function*: that there should be fidelity to the key goals or *functions* of an evidence-based program [e.g., reinforce quality implementation (36)]. But that the specific *forms* of activities to address these functions should be tailored to specific contextual factors. Public health has always been sensitive to the need to adapt to cultural and local community factors using approaches such as community-based participatory research (37), but DIS extends this focus on adaptations across the lifespan of a program and to address adaptations to the EBP, the strategies used to implement the program, and the context itself (16). DIS authors have also focused on the need for adaptations to address issues of health equity (8) and for programs to be sustainable (11).

Aging and public health research needs to be much more rapid than it has traditionally been to be relevant to decision makers and community groups, to respond to rapidly changing context, and to contribute to learning health systems. One active area of DIS focuses on how to speed the application and relevance of dissemination and implementation research (38, 39). It is acknowledged that research must be not only Rapid, but also Relevant to community and clinical partners, Rigorous, attend to Resources Required, and Replicable [the 5 Rs (40)]. With context continually changing, it is usually the case that adaptions need to be iterative and there is active D&I research applying D&I frameworks in ways that are rapid and iterative (41, 42).

### Begin with the end in mind

It may seem obvious but DIS is best conceptualized by initiating action with an eye toward what outcomes are intended. In the early DIS work there was focused on the concept of designing for dissemination (43). It included thinking about the characteristics of interventions that may be most likely to be adopted in typical service settings and to plan for dissemination from the outset of a project. This concept of designing for dissemination has been expanded and we recommend aging and public health researchers interested in DIS take the approach of designing for dissemination, equity, and sustainability (44). This work often includes careful selection of delivery system partners to ensure there is a broad representation in, for example, senior-serving settings, to ensure that those providing services for populations experiencing health disparities and inequities contribute to intervention and implementation strategy design decisions (45). Theory is, again, important in the process of designing for dissemination, equity, and sustainability. For example, considering the ideal or preferred intervention characteristics using PRISM contextual factors focused on participant and organizational perspectives can result in interventions that are attractive to underserved audiences and aligned with the assets available in the organizations that serve that audience (46).

### The need for de-implementation

While DIS focused initially on the getting evidence-based interventions into practice, there is also need for the de-implementation of low value interventions that that may be either ineffective or harmful (47). De-implementation and de-intensification represent a growing area of DIS in aging and public health in which new theories and methods may be needed to reduce the use or overuse of ineffective or harmful interventions (19). To help providers decide when and how to “de-adopt” treatments in patients with complex needs. Indeed, the removal of care that may be perceived as potentially beneficial by older patients could be especially challenging for de-implementation (19). User-centered approaches that include patients and providers in the identification of pathways toward de-implementation or de-intensification may be promising approaches, particularly for older adults (48).

## Conclusion

Focusing on the what, when, how, and why of dissemination and implementation will advance the speed of translation, as well as the broader public health impact, of evidence-based interventions in aging and public health. We note that this brief article is necessarily cursory and several other DIS recommendation and guidance documents exist for scientists and practitioners that also include many more useful examples. In addition, we used RE-AIM and PRISM as our examples of outcomes and contextual DIS frameworks, though there are a myriad of other frameworks from which to choose. As with our recommendations for matching context-strategy-mechanisms-outcomes, we encourage those in aging and public health research to investigate what is available and pick what seems to be the best fit for your research question and context. There are great resources such as www.dissemination-implementation.org and Brownson et al. (49) that can help facilitate framework selection. Additionally, we invite researchers and practitioners to engage with the National RE-AIM Workgroup and visit (www.re-aim.org) to learn more about public health approaches to improving population health across the life course.

## Author contributions

PE and RG collaboratively conceptualized this paper. Both authors contributed to writing sections of the initial draft and subsequent revisions leading to the final submitted manuscript.
